# Isolated positive anti-SS-B autoantibodies are not related to clinical features of systemic autoimmune diseases: Results from a routine population survey

**DOI:** 10.1371/journal.pone.0185104

**Published:** 2017-09-20

**Authors:** Sabine Jardel, Nicole Fabien, Arnaud Hot, Sandra Vukusic, Jacques Tebib, Vincent Cottin, Pascal Sève, Maurice Laville, Alexandre Belot, Isabelle Durieu, Lorna Garnier, Frédéric Coutant, Quitterie Reynaud, Jean Christophe Lega

**Affiliations:** 1 Department of Internal and Vascular Medicine, Centre Hospitalier Lyon Sud, Hospices Civils de Lyon, Claude Bernard University Lyon 1, University of Lyon, Lyon, France; 2 Department of Immunology, Centre Hospitalier Lyon Sud, Hospices Civils de Lyon, Lyon, France; 3 Department of Internal Medicine and Vascular Medicine, Centre Hospitalier Edouard Herriot, Hospices Civils de Lyon, Claude Bernard University Lyon 1, University of Lyon, Lyon, France; 4 Department of Neurology A, Hôpital Neurologique Pierre Wertheimer, Hospices Civils de Lyon, Claude Bernard University Lyon 1, University of Lyon, Lyon, France; 5 Department of Rheumatology, Centre Hospitalier Lyon Sud, Hospices Civils de Lyon, Claude Bernard University Lyon 1, University of Lyon, Lyon, France; 6 National Reference Centre for Rare Pulmonary Diseases, Department of Respiratory Medicine, Louis Pradel Hospital, Hospices Civils de Lyon, UMR 754, Claude Bernard University Lyon 1, University of Lyon, Lyon, France; 7 Department of Internal Medicine, Hôpital de la Croix Rousse, Hospices Civils de Lyon, Claude Bernard University Lyon 1, University of Lyon, Lyon, France; 8 Department of Nephrology, Hospices Civils de Lyon, Centre Hospitalier Lyon Sud, Hospices Civils de Lyon, Claude Bernard University Lyon 1, University of Lyon, Lyon, France; 9 Pediatric Nephrology, Rheumatology, Dermatology Unit, Hospices Civils de Lyon, INSERM U1111, Hôpital Femme Mère Enfant, Hospices Civils de Lyon, Claude Bernard University Lyon 1, University of Lyon, Lyon, France; 10 UMR 5558, Laboratoire de Biométrie et Biologie Evolutive, CNRS, Claude Bernard University Lyon 1, University of Lyon, Lyon, France; Keio University, JAPAN

## Abstract

**Objective:**

To assess in clinical practice the frequency and diagnosis associated with the SS-B-positive/SS-A negative autoantibody profile.

**Methods:**

We analyzed a one-year consecutive population of 624 patients referred by clinicians to the immunology laboratory to investigate anti-SS-A and/or anti-SS-B autoantibodies, who were detected using luminex technology. Data were analyzed for patients with isolated anti-SS-B autoantibodies. The clinical characteristics and diagnosis of connective tissue diseases (CTD) were retrieved according to the international criteria.

**Results:**

Among 1173 sera positive for anti-SS-A and/or anti-SS-B autoantibodies from 624 patients, we identified 84 patients (13.5%) that had isolated anti-SS-B. Among the 75 patients positive for anti-SS-B with known clinical data, 15 were diagnosed with a CTD (20%) including 4 systemic lupus erythematosus (5%), 4 rheumatoid arthritis (5%), 2 idiopathic inflammatory myositis (3%), 1 primary Sjögren’s syndrome pSS (1%), 1 systemic sclerosis (1%), 2 undefined CTD (3%), and 1 mixed CTD (1%). Among the 60 other patients, 18 had non-CTD autoimmune diseases and 42 had non-autoimmune diseases. Within the CTD population, the presence of isolated anti-SS-B was not significantly associated to characteristic indicating a specific syndrome. There was no association between diagnosis of CTD and level of anti-SS-B autoantibodies (p = 0.70). Arthralgia was the more frequent sign and encountered in 10 patients (67%), of whom 3 had arthritis.

**Conclusion:**

The presence of anti-SS-B, without anti-SS-A autoantibodies using luminex technology, was not associated with CTD, especially pSS, in daily clinical practice. Our data suggests that the SS-B serological profile is not contributive for the classification criteria of pSS.

## Introduction

Anti-SS-A(Ro) and anti-SS-B(La) autoantibodies are encountered in 50 to 70% of patients with primary Sjogren’s Syndrome (pSS) [1], 20–50% of patients with systemic lupus erythematosus SLE [2], and 5% of patients with rheumatoid arthritis (RA) [2]. Two profiles of anti-SS-A/anti-SS-B reactivity are described in pSS, the combined anti-SS-A/anti-SS-B positivity and anti-SS-A positivity alone, with the former being more common than the latter. Patients with pSS and positive for both anti-SS-A and anti-SS-B autoantibodies have more severe glandular involvements and extra-glandular manifestations compared to seronegative patients [1,3]. The value of these autoantibodies in other connective tissue disease (CTD) is still debated. Isolated anti-SS-B positivity was reported as suggestive of cutaneous lupus and pSS in one series of patients [4]. In a recent study, anti-SS-B, with or without anti-SS-A, was significantly associated with lymphoma in patients with pSS [5]. However, a report from an international cohort of 3297 pSS patients questioned the value of isolated anti-SS-B in the description of clinical profile and in morbidity [3], leading to the exclusion of this criteria of the recent pSS classification [6].

The present study was therefore undertaken to assess the frequency and diagnosis associated with isolated SS-B-positive autoantibody profile in a one-year consecutive cohort of 624 patients referred by clinicians to the immunology laboratory for evaluation of such autoantibodies.

## Methods

### Patient selection

We looked for consecutive positive serum for anti-SS-A autoantibodies and/or anti-SS-B autoantibodies, detected between January 2013 and January 2014 in the laboratory of immunology of the Hospices Civils de Lyon. These samples were mainly referred by in house internists (56%), rheumatologists (16%), nephrologists (9%), neurologists (8%), dermatologists (4%) and pulmonologists (1%). Clinical data were analyzed for patients with isolated anti-SS-B autoantibodies. French observational studies from data obtained from a retrospective study without any additional therapy or monitoring procedure do not need informed consent for the patients as anti-SS-B antibodies were analyzed as part of diagnostic investigation. This work was declared at the Commission Nationale de l'Informatique et des Libertés (CNIL). One author had access to identifying information, but patient records and information were anonymized and de-identified prior to analysis.

### Diagnostic criteria for connective tissue disease

Connective tissue diseases were diagnosed according to international criteria. 2012 criteria of the Systemic Lupus International Collaborating Clinics was used for SLE [7], 2010 classification criteria of the collaborative American College of Rheumatology (ACR) and European League Against Rheumatism (EULAR) for RA [8], 2013 classification criteria of the collaborative ACR/EULAR for systemic sclerosis (SSc) [9], Sharp criteria for mixed connective tissue disease [10], revised international criteria of the American-European consensus group for pSS [11], 119th European NeuroMuscular Centre international workshop for idiopathic inflammatory myositis (IIM) [12]. Undifferentiated CTD was diagnosed if objective criteria were present, but not sufficient for the diagnosis of overt CTD, in addition to positive antinuclear autoantibodies (ANA) [13].

### Patient analysis and laboratory test

We retrospectively collected clinical and biological data using the database of the Department of Immunology of Hospices Civils de Lyon. Demographic, clinical and biological data were retrieved through a retrospective chart review.

Minor salivary gland biopsy was performed only if there were clinical signs evocative of SS such as xerostomia, and was evaluated by an expert histopathologist. Positive threshold was a focus score>1 [11]. Xerophtalmia was evaluated using the Schirmer’s test, which was positive when inferior to 5 mm in 5 minutes.

Antinuclear autoantibodies were tested by an indirect immunofluorescence technique with HEp2 cells (Biorad, Marnes la Coquette, France). Anti-SS-A and anti-SS-B autoantibodies were analyzed by a Luminex technology with the manufacturer’s threshold of positivity at 1 Antibody Index (AI) (Bioplex 2200, Biorad, Marnes la Coquette, France). Anti-nucleosome, anti-U1-RNP, anti-Sm, and anti-JO1 were analyzed with the same technique. Anti-PM/Scl, anti-PL-7, anti-PL12, anti-EJ, anti-OJ, anti-Mi2, anti-Ku autoantibodies were analyzed using an immunodot technique (Euroimmun, Bussy-St-Martin, France). Anti-fibrillarine autoantiboy was analyzed by UNICAP (Thermofischer, Freiburg, Germany). Anti-double stranded (dsDNA) autoantibodies were detected by radioimmunoassay (Trinity Biotech, Siemens, France). Anti-CCP antibodies and rheumatoid factor (RF) were detected using a Luminex technology (Bioplex) and a nephelometry technique (Dade Behring), respectively.

### Data analysis

Baseline characteristic were assessed by descriptive statistics. Continuous variables were compared using t-tests or non-parametric equivalent. Categorical variables were compared using the chi-squared statistic or Fisher’s exact test. For all statistical analyses, p<0.05 was considered significant. Analyses were performed with R (R Foundation for Statistical Computing, Vienna, Austria).

## Results

**Fig 1:**
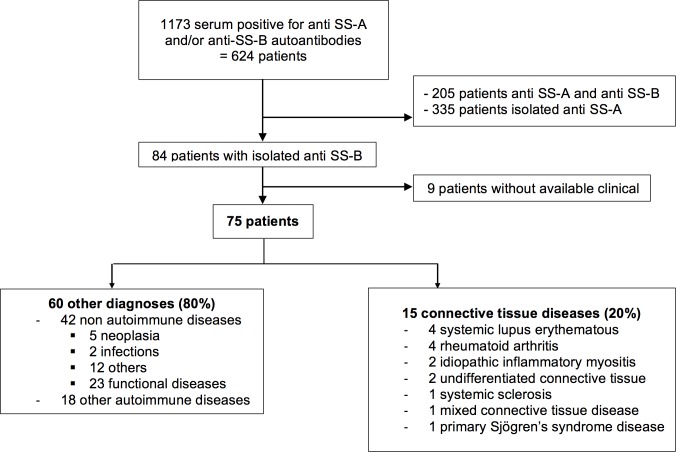
Retrospective chart review of patients.

One thousand one hundred and seventy-three sera from 624 patients were positive for anti-SS-A and/or anti-SS-B autoantibodies between January 2013 and January 2014. Two hundred and five patients (32.8%) were positive for both anti-SS-A and anti-SS-B, and 335 (53.7%) were positive for anti-SS-A autoantibodies only. Eighty-four patients (13.5%) were positive only for anti-SS-B autoantibodies. No clinical data was available for 9 patients (Fig 1). The final analysis included 75 patients. The median age of these patients was 50 years (range 3–91 years); and 80% were female.

### Clinical data of 75 patients with isolated anti-SS-B autoantibodies

Fifteen adult patients (20%) suffered from CTD with SLE in 4 patients (5%), RA in 4 patients (7%), IIM in 2 patients (3%), pSS in 1 patient (1%), SSc in 1 patient (1%), undefined CTD in 2 patients (3%) and mixed CTD in 1 patient (1%) (Table 1).

**Table 1:** Distribution of diagnosis according to connective tissue disease, non-connective tissue disease autoimmune and non-autoimmune diseases in patients with isolated anti-SS-B autoantibodies.

CTD	N = 15	Other autoimmune disease	N = 18	Other non autoimmune disease	N = 42
Systemic lupus erythematous	4	**Other systemic autoimmune rheumatic diseases**	**7**	**Neoplasia**	**5**
Rheumatoid arthritis	4	Relapsing polychondritis	1	Lymphoma	3
Idiopathic inflammatory myositis	2	Psoriatic rheumatism	1	Lung	1
Undifferentiated connective tissue disease	2	ANCA associated vasculitis	3	Breast	1
Systemic sclerosis	1	Coeliac disease	1	**Infection**	**2**
Primary Sjögren’s syndrome	1	Juvenile idiopathic arthritis	1	**Other diseases**	**12**
Mixed connective tissue disease	1	Interstitial lung disease	2	Post-infectious acute glomerulonephritis	1
		Vogt-Koyanagi-Harada syndrome	1	Polycythemia vera	1
		Localized scleroderma	1	Erythropoietic porphyria	1
		Acute disseminated encephalomyelitis	1	Extrinsic allergic alveolitis	1
		Multiple sclerosis	3	Langerhans-Cell histiocytosis	1
		Auto-immune hepatitis	1	Amyotrophic lateral sclerosis	1
		Antiphospholipid syndrome	1	Cystic fibrosis	1
		Idiopathic uveitis-meningitis	1	Migraine headache	1
				Inherited collagen disease	1
				Immuno-allergic acute renal insufficiency	1
				Eosinophilic asthma	1
				**Functional disorder**	**23**

Sixty patients (80%) were not diagnosed as CTD (Table 1). Eighteen (24%) had non-CTD autoimmune diseases; 42 patients (56%) were diagnosed as non-autoimmune diseases of whom 23 (31%) had functional disorders without clear general or visceral involvement.

Characteristic of the 15 patients with CTD are presented in Table 2. Arthralgia were the most frequent symptoms in 10 patients (67%), of whom 3 had arthritis, predominantly in SLE and RA patients. Seven patients (47%) had Raynaud's phenomenon. No death occurred during the follow-up.

**Table 2:** Clinical and biologic characteristics of patients diagnosed as connective tissue disease.

Case	Age (yrs)	Sex	Anti-SS-B (AI)	ANA titer	Fluorescence type	Other autoantibodies	Connective tissue disease feature	CTD
1	79	F	7.9	1280	speckled	dsDNA, nucleosome	cutaneous lupus, alopecia, arthralgia	SLE
2	51	F	1.2	1280	speckled	-	arthralgia, leukopenia, thrombocytopenia	SLE
3	39	F	>8.0	160	diffuse	dsDNA, nucleosome	arthralgia, xerostomia, complement consumption, cyoglobulinemia	SLE
4	26	F	1.2	320	speckled	RF, CCP	oral ulceration, arthralgia, RP, leukopenia, APL	SLE
5	74	F	1.1	1280	diffuse	-	muscular involvement, CK	PM
6	72	M	1.2	1280	diffuse and speckled	U1RNP	specific DM rash, CK, cholestasis	DM
7	45	F	1	<160	-	CCP	arthritis, pericarditis	RA
8	75	F	4.4	320	diffuse	CCP	arthralgia, RP, leucopenia	RA
9	59	F	2.8	640	speckled	dsDNA	arthritis	RA
10	44	F	1.6	<160	-	CCP	arthritis, RP	RA
11	47	F	1	1280	centromere	ACA	arthralgia, RP, sclerodactyly, digital ulceration, SP-NP	SSc
12	35	F	3.4	1280	centromere	nucleosome, ACA	RP, malar rash	UCTD
13	47	F	1.7	1280	speckled	-	digital ulceration, ILD, SP-NC	UCTD
14	50	F	4.4	1280	speckled & centromere	nucleosome, Sm, ACA	arthralgia, SP-NC, RP	Sharp
15	69	F	1.1	320	diffuse	Ku, fibrillarine, EJ, PM/Scl, PL7	xerostomia, MSGB focus score >1, motor and sensory axonal polyneuropathy, RP, SP-NC	pSS

ACA: anti-centromere autoantibodies, AI: antibody indexAPL: antiphospholipid antibodies, CCP: anticyclic citrullinated peptide antibodies, CK: creatine kinase elevation, DM: dermatomyositis, dsDNA: double stranded DNA, F: female, ILD: interstitial lung disease, M: male; MSGB: minor salivary gland biopsy, PM: polymyositis, pSS: primary Sjögren’s syndrome, RA: rheumatoid arthritis, RF: rheumatoid factor, RP: Raynaud phenomenon, SP-NC: scleroderma pattern at nailfold capillaroscopy, SLE: systemic lupus erythematous, SSc: systemic sclerosis, yrs: years

Among the 4 SLE patients, articular (4 patients) involvement was predominant. One patient had cutaneous involvement. No patient had renal or neurologic manifestations. Three patients were treated by hydroxychloroquine and corticosteroids. One patient was treated by anti-tumor necrosis factor TNF therapy. None of the patients required further immunosuppressive therapy. Two out of 4 were positive for anti-dsDNA autoantibodies, 1 for RF and anti-CCP autoantibodies.

Four cases of RA were diagnosed, with typical symmetric and bilateral arthritis. One RA patient experienced an episode of uncomplicated pericarditis treated by aspirin. All RA patients received methotrexate, 2 were treated with anti-TNF.

The patient with pSS experienced extraglandular manifestations with axonal polyneuropathy. One patient had a dermatomyositis and was positive for anti-U1RNP autoantibodies, and the other one suffering for unclassified IIM had muscular weakness, and creatine kinase elevation, without available muscular biopsy.

One patient had limited cutaneous SSc, positive for anti-centromere autoantibodies. The diagnosis of undefined CTD was retained for 2 patients: one patient had a gestalt evocative of SSc with sclerodactily and interstitial lung disease, another patient presented incomplete SLE encompassing malar rash, Raynaud’s phenomenon, anti-phospholipid autoantibodies, and anti-centromere autoantibodies.

### Factors associated with connective tissue disease

**Fig 2:**
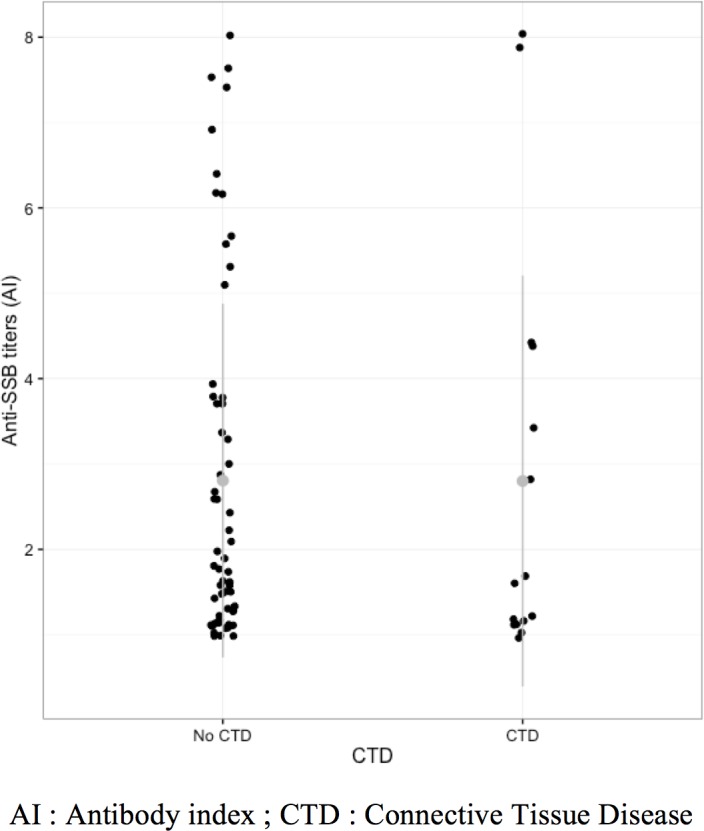
Distribution of anti-SSB titers according to the diagnosis.

**Fig 3:**
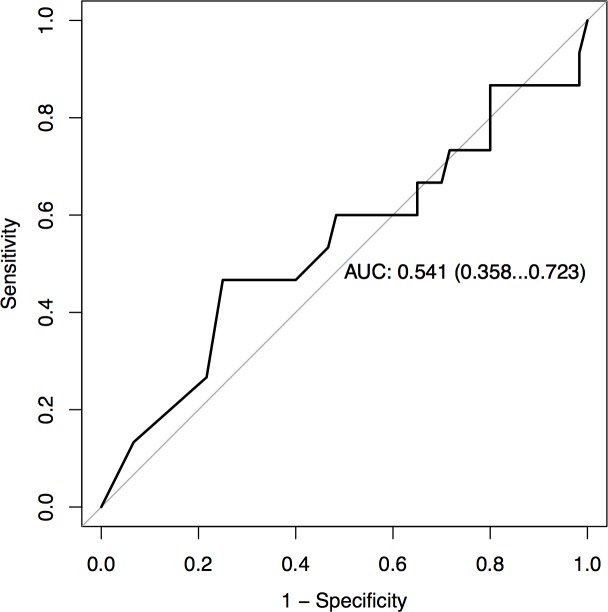
Receiver operating characteristics (ROC) curve of anti-SS-B titers for the diagnosis of connective tissue disease in the 75 positive patients.

Among the 75 patients positive for isolated anti-SS-B autoantibodies, 31 (41%) had positive ANA defined as a titer superior to 1/160. The CTD patients had significantly more ANA than non-CTD patients (87% versus 30%; OR 13.9, 95% CI 2.7–139.7) (Table 3). The fluorescence pattern was diffuse or centromere in 8 (53%) CTD patients. Low titers between 1 and 2 AI of anti-SS-B autoantibodies were detected in both non-CTD (32 [53%] of 60 patients of anti-SS-B) as well as in CTD (9 [60%] of 15 patients) (p = 0.78). CTD diagnosis was correlated with the positivity for other anti-extractable nuclear antigens anti–ENA autoantibodies (3 anti-centromere and 1 anti-U1-RNP), (33% versus 0%, p<0.0005), anti-CCP autoantibodies (27% versus 2%, p = 0.005), anti-dsDNA autoantibodies (20% versus 2%, p = 0.03), and anti-nucleosome autoantibodies (27% versus 0%, p = 0.001). There was no correlation between diagnosis of CTD and level of anti-SS-B autoantibodies (p = 0.70), age (p = 0.51), or sex (p = 0.28). Distribution of anti-SS-B titers according to diagnosis is represented in Fig 2. The area under the curve for the CTD diagnosis according to the level of anti-SS-B antibodies was 0.54 (95%CI 0.36–0.72) by ROC analysis (Fig 3).

**Table 3:** Factors associated with the diagnosis of connective tissue disease in patients positive for isolated anti-SS-B autoantibodies.

	CTD	Non CTD	Univariate analysis
	(n = 15)	(n = 60)	p-value
Age (year, mean ±SD)	54.1 ±16.3	50.8 ±18.5	0.51
Male sex	1 (7%)	14 (23%)	0.28
ANA titer ≥160	13 (87%)	18 (30%)	<0.0005
Anti-SS-B aAbs titers (mean [SD]) (AI)	2.8 ±2.4	2.8 ±2.1	0.6
Other autoantibodies	7 (47%)	1 (2%)	<0.0001
Other anti-ENA aAbs	5 (33%)	0 (0%)	<0.0005
Anti-CCP aAbs	4 (27%)	1 (2%)	0.005
Rheumatoid factor	2 (133%)	2 (3%)	0.18
Anti-dsDNA aAbs	3 (20%)	1 (2%)	0.03
Anti-nucleosome aAbs	4 (27%)	0 (0%)	0.001

ANA: antinuclear autoantibodies, aAbs: autoantibodies, AI: antibody index, CCP: cyclic citrullinated peptide, CTD: connective tissue disease, dsDNA: double stranded DNA, IQR: interquartile range, SD: standard deviation

## Discussion

To our knowledge, our study is the largest consecutively cohort addressing the clinical value of isolated anti-SS-B-positive autoantibodies in daily practice.

Anti-SS-B autoantibodies were initially described invariably accompanied by anti-SS-A autoantibodies [14], and their respective pathogenic role was difficult to ascertain [14]. In the 90’s, a study demonstrated that anti-SS-B autoantibodies had a high diagnostic specificity for Sjögren’s syndrome [15], and the European preliminary classification published in 1993 proposed the presence of anti-SS-A and/or anti SS-B as a diagnostic criteria [16]. In a recent study, anti-SS-B autoantibodies, with or without anti-SS-A, have been significantly associated with the presence of lymphoma in patient with pSS [5]. Nevertheless, the meaning of isolated anti-SS-B autoantibodies is not really clearly demonstrated, neither clinically nor as a prognostic factor.

The prevalence of such isolated autoantibodies among anti-SS-A/SS-B patients can be variable according to the methods of detection [3,17]. We found a prevalence of 12%, which is similar to a previous series using 2 different techniques, i.e immunodiffusion and line immunoassay [4]. It appears therefore that such immunologic situation is not rare, and thus questions about the signification of isolated anti-SS-B autoantibodies in daily clinical practice.

In our study, 40% of patients with isolated anti-SS-B had positive ANA, but patients with CTD had significantly more often positive ANA than patients without CTD. One interpretation could be that anti-SSB sera with a positive ANA had another autoantibody that was not detected by the limited autoantibody analysis performed in this study. We therefore found here that the clinical value of isolated anti-SS-B autoantibodies was strongly related to the positivity of ANA by indirect immunofluorescence technique. By consequence, we can suggest that searching anti-SS-B autoantibodies in case of ANA negativity is not necessary. This was also suggested by a previous study exploring the clinical value of anti-ENA despite negative ANA, using an ELISA technique [18]. This study included 11 patients with isolated anti-SS-B of whom only 2 patients were diagnosed as suffering from CTD, namely pSS. This statement is reinforced by the lack of key phenotypic features in patients with CTD and SS-B positive/SS-A negative auto-antibodies. In fact, our series clearly showed that the presence of isolated anti-SS-B autoantibodies is not associated to any specific diagnosis, general characteristics or organ involvement. Recently, Baer et al [3] published a series of 3297 patients of the Sjögren’s International Collaborative Clinical Alliance SICCA cohort, in which 74 were anti-SS-B alone using the same luminex technique as ours. They showed that participants with anti-SSB alone were comparable to those with negative SS-A/SS-B serology for the SS key phenotypic features. Moreover, and contrary to a previous series with similar design but using an immunodiffusion technique [4], we did not confirm an association between cutaneous lupus or pSS and isolated anti-SS-B autoantibodies. The recent classification of the ACR and American-European Consensus Group for pSS [6] has excluded anti-SS-B autoantibodies positivity. Given the absence of phenotypic feature associated with isolated anti-SS-B autoantibodies, and the low negative and positive likelihood ratio in diagnosis of pSS, our results support this exclusion, as suggested by Baer et al [3].

Our series emanates from a single laboratory of autoimmunity that centrally collects samples from numerous departments of all medical specialties from public university hospitals in Lyon, recruiting from a population of more than 1.4 million inhabitants. Thus, we believe that we were certainly covering all the fields of systemic or organ specific autoimmune diseases. However, our study had some limitations, such as the cross sectional design that might have underscored the estimated prevalence of CTD despite our effort in reclassification after review of medical charts. Indeed, evidence has been reported that patients with undifferentiated CTD and antibodies to SS-A can progress in a relatively short period to overt CTD diseases [19]. The retrospective nature of the study may introduce information bias. Finally, in regards of the low prevalence of visceral involvement in our cohort, we could not exclude that isolated anti-SS-B autoantibodies were a marker of CTD with good prognosis.

In conclusion, the present study demonstrates that the positivity of anti-SS-B using luminex technology, without anti-SS-A autoantibodies, has little association with CTD including pSS in daily clinical practice, especially in case of ANA negativity. Given the lack of predictive value of organ involvement related to CTD and the absence of correlation between anti-SS-B autoantibody titers and CTD diagnosis, the isolated positivity of anti-SS-B autoantibodies does not provide additional clinical support in medical decision. This serological profile must thus not be misinterpreted. The results of the present series advocate in favor of the decision of the Rheumatology/European League Against Rheumatism Classification Criteria classification to omit anti-SS-B for the diagnosis of pSS.

## Supporting information

S1 Dataset Dataset of included patients. (XLSX)
